# Inhibin B and anti-Müllerian hormone as markers of gonadal function after hematopoietic cell transplantation during childhood

**DOI:** 10.1186/1471-2431-11-20

**Published:** 2011-02-25

**Authors:** Sylvie Laporte, Ana-Claudia Couto-Silva, Séverine Trabado, Pierre Lemaire, Sylvie Brailly-Tabard, Hélène Espérou, Jean Michon, André Baruchel, Alain Fischer, Christine Trivin, Raja Brauner

**Affiliations:** 1 Université Paris Descartes and AP-HP, Hôpital Bicêtre, Unité d'Endocrinologie Pédiatrique, 94270 Le Kremlin Bicêtre, France; 2 State of Bahia, Center for Diabetes and Endocrinology (CEDEBA), Bahia, Brazil; 3 AP-HP, Hôpital Bicêtre, Laboratoire de Génétique moléculaire, Pharmacogénétique et Hormonologie, and Univ Paris Sud, 94270 Le Kremlin Bicêtre, France; 4 G-SCOP, INP-Grenoble, UJF, CNRS, 38000 Grenoble, France; 5 AP-HP, Hôpital Saint Louis, Service d' Hématologie-Greffes de Moelle, 75475 Paris, France; 6 Institut Curie, Département d'Oncologie Pédiatrique, 75248 Paris, France; 7 AP-HP and Université Paris7 Paris-Diderot, Hôpital Robert Debré, Service d'Hématologie Pédiatrique, 75019 Paris, France; 8 Université Paris Descartes and AP-HP, Hôpital Necker-Enfants Malades, Unité d'Immunologie, Hématologie et Rhumatologie Pédiatriques, 75743 Paris, France; 9 AP-HP, Hôpital Necker-Enfants Malades, Service d'Explorations Fonctionnelles, 75743 Paris, France

## Abstract

**Background:**

It is difficult to predict the reproductive capacity of children given hematopoietic cell transplantation (HCT) before pubertal age because the plasma concentrations of follicle-stimulating hormone (FSH) and luteinizing hormone (LH) are not informative and no spermogram can be done.

**Methods:**

We classified the gonadal function of 38 boys and 34 girls given HCT during childhood who had reached pubertal age according to their pubertal development and FSH and LH and compared this to their plasma inhibin B and anti-Müllerian hormone (AMH).

**Results:**

Ten (26%) boys had normal testicular function, 16 (42%) had isolated tubular failure and 12 (32%) also had Leydig cell failure. All 16 boys given melphalan had tubular failure. AMH were normal in 25 patients and decreased in 6, all of whom had increased FSH and low inhibin B.

Seven (21%) girls had normal ovarian function, 11 (32%) had partial and 16 (47%) complete ovarian failure. 7/8 girls given busulfan had increased FSH and LH and 7/8 had low inhibin B. AMH indicated that ovarian function was impaired in all girls.

FSH and inhibin B were negatively correlated in boys (P < 0.0001) and girls (P = 0.0006). Neither the age at HCT nor the interval between HCT and evaluation influenced gonadal function.

**Conclusion:**

The concordance between FSH and inhibin B suggests that inhibin B may help in counselling at pubertal age. In boys, AMH were difficult to use as they normally decrease when testosterone increases at puberty. In girls, low AMH suggest that there is major loss of primordial follicles.

## Background

Conditioning for hematopoietic cell transplantation (HCT) may alter the production of gonadal hormones (testosterone in boys, estradiol and progesterone in girls) and the viability of the germ cells. Gonadal failure may result in incomplete sexual development and growth at puberty, and sterility in adulthood. Thus, gonadal hormones are required for the development of secondary sexual characteristics and the growth spurt which normally occurs at puberty.

There are few reports of patients given HCT during childhood being fertile (2 boys and 3 girls in Salooja [1], 2 boys and 9 girls in Sanders [2]). Sanders et al [2] showed that 15 (13%) of 114 prepubertal boys developed normal testicular function and the partners of 2 of them became pregnant; the great majority of those who recovered testicular function had been given cyclophosphamide without irradiation. In parallel, 23 (28%) of 82 prepubertal girls developed normal ovarian function, 9 of whom became pregnant; the pregnancies of all 5 given total body irradiation (TBI) ended in spontaneous abortion.

It is difficult to predict the reproductive capacity of a child before pubertal age because the plasma concentrations of follicle-stimulating hormone (FSH) and luteinizing hormone (LH) are not informative and no spermogram can be done. The plasma concentrations of inhibin B and anti-Müllerian hormone (AMH) might be helpful at this age. In boys, inhibin B is produced by the Sertoli cells. Its plasma concentration is the best plasma marker of spermatogenesis [3-5]. A study of 218 subfertile men found that their inhibin B concentration accurately (95%) differentiated between competent and impaired spermatogenesis, while the FSH concentration was less accurate (80%) [5]. In girls, inhibin B is produced only by the granulosa cells of small antral follicles, while AMH is produced by the granulosa cells of pre-antral follicles, i.e. the ovarian reserve. Its plasma concentration decreases as the number of follicles decreases with age, with a strong correlation between age at menopause and AMH measured between the ages of 20 and 36 years [6].

We evaluated the gonadal function of 72 patients given HCT during childhood after they had reached pubertal age. The objectives were: 1) to compare the evaluation of gonadal function by pubertal stage, testicular volume in boys and menstrual pattern in girls, plasma FSH and LH concentrations to the plasma concentrations of inhibin B and AMH; 2) to evaluate the influence of the age at HCT, and the interval between HCT and evaluation, conditioning and, for chemotherapy, the use of cyclophosphamide, busulfan and melphalan on gonadal function.

## Methods

### Patients

This retrospective single center study included 72 patients (38 boys, 34 girls) given HCT between 1976 and 2006 (median 1991, Table 1) and followed by one of us (R. Brauner) in a university pediatric hospital. The boys were younger than 15 years (8.2 ± 0.6 yr) at HCT and the girls younger than 13 years (7.0 ± 0.6 yr). Puberty began in 3 boys and 2 girls at HCT. They had reached pubertal age (over 13 years for boys and 11 years for girls) when their gonadal function was evaluated. The interval between HCT and evaluation was 8.3 ± 0.6 yr in boys and 6.9 ± 0.6 yr in girls.

**Table 1:** Patient characteristics

	Boys (n = 38)	Girls (n = 34)	Total
Age at HCT, yr	8.2 ± 0.6	7.0 ± 0.6	
	(1.0-15)	(0.6-13)	
Age at evaluation, yr	16.5 ± 0.3	13.9 ± 0.3	
	(13.2-21.3)	(11-17.3)	
Interval between HCT and evaluation, yr	8.3 ± 0.6	6.9 ± 0.6	
	(1.1-16.0)	(1.5-14.4)	
Initial disease (n)			
Malignant	35	26	61
Non malignant	3	8	11
Conditioning, n			
TBI	34	24	58
TLI	3	3	6
Chemotherapy alone	1	7	8
mean ± se (range)			

The initial diseases were malignant [acute lymphoblastic leukemia (n = 31), acute myeloid leukemia (n = 16), chronic myelogenous leukemia (n = 3), lymphoma (n = 5), neuroblastoma (n = 5), nephroblastoma (n = 1)], or non-malignant [severe aplastic anemia (n = 6), congenital immunodeficiency (n = 4), myelodysplasia (n = 1)]. Of the patients with malignant disease, 34 were given HCT in first remission and 27 in second or third remission. The patients were allografted (70%) or autografted (30%). The conditioning protocol for HCT included chemotherapy in all, TBI 12 Grays (Gy) as six fractions of 2 Gy over 3 consecutive days, or a single dose 10 Gy TBI, 5 or 6 Gy total lymphoid irradiation (TLI) as single 4-h exposures or chemotherapy alone (Table 1). The total chemotherapy doses were cyclophosphamide (120, 150 or 200 mg/kg according to the disease), melphalan (140 mg/m^2^) and/or busulfan (600 mg/m^2^). The other drugs were cytarabine in 12 (18 or 24 g/m^2^), etoposide in 5 (400 mg/m^2^), methotrexate in 4 and vincristine in 5.

Fifteen other boys and 11 girls were excluded because no samples were available for measuring inhibin B. Their characteristics were similar to those who were included. Another 75 patients seen during the same period and fulfilling these criteria were excluded because they had factors other than conditioning for HCT that might have interfered with their gonadal function: the initial disease [Fanconi's anemia (n = 19), Blackfan-Diamond anemia (n = 3), thalassemia (n = 3), drepanocytosis (n = 2), Seckel disease (n = 1)], central nervous system involvement or additional irradiation (n = 47).

### Protocol

Informed consent for evaluation and treatment was obtained from the patients and their parents. The Ethical Review Committee (Comité de Protection des Personnes Ile de France III) stated that ''This research was found to conform to generally accepted scientific principles and research ethical standards and to be in conformity with the laws and regulations of the country in which the research experiment was performed".

We recorded testicular dimensions [7] and pubic hair development in the boys [8]. Because the testicular dimensions may be altered by the tubular failure caused by the conditioning protocol, the pubertal stage was defined by the pubic hair development and the plasma testosterone concentration: P1 - below 0.5 ng/mL, P2 - 0.5-2 ng/mL, P3 - 2-3 ng/mL and P4-5 over 3 ng/mL [adapted from 9]. We recorded the age at breast development and the occurrence and progress of their menstruations in the girls [10]. Plasma samples were collected before the substitutive treatment except in 5 girls in whom the estradiol treatment was interrupted for at least 2 months (see below), and a long time after graft versus host disease. We measured the basal plasma concentrations of FSH, LH and testosterone in boys and estradiol in girls. Aliquots of plasma were frozen at -20°C and used to measure concomitant plasma inhibin B (in all) and AMH (31 boys and 25 girls) concentrations. We used the last sample taken from patients who had undergone more than one laboratory evaluation.

Normal gonadal function was defined by the occurrence of spontaneous puberty in both sexes, plus regular menstruations in girls, and normal basal plasma FSH (< 9 IU/L) and LH (< 5 IU/L) concentrations. In boys, tubular failure was defined by an increased plasma FSH concentration, and Leydig cell failure by an increased plasma LH concentration with a normal (partial failure) or low (complete failure) plasma testosterone concentration. The normal basal testosterone concentration in adult boys is 3.5-8.5 ng/mL. In girls, ovarian failure was defined by increased plasma FSH and/or LH concentrations, which is partial when pubertal development is spontaneous and plasma estradiol is normal, and complete when pubertal development is partial or absent and plasma estradiol low.

Two boys with partial and the one with complete Leydig cell failure were given testosterone heptylate (25 mg i.m. every 14 days) at the age of around 13 years. Seven girls with partial and the 16 with complete ovarian failure were given oral ethinyl estradiol (2 μg/day) at the age of around 12 years. The doses were increased to adult levels, and associated with cyclical progestin in girls when they had finished growing.

The growth hormone (GH) secretion of the patients given TBI who had a decreased growth rate was evaluated by a stimulation test. The test was repeated in those with a low peak to decide on GH treatment, and in those whose growth rate remained low despite a normal GH peak [11]. Twenty-four patients were given GH. Plasma cortisol and prolactin concentrations were normal in all patients. The 32 patients with high plasma thyroid stimulating hormone concentrations after TBI or TLI were given thyroxin (50 μg/m^2^/day).

### Methods

When the assay method for a given hormone was changed during the study period, it was cross-correlated with the earlier method. Thus, the results are comparable throughout the whole period.

The plasma concentrations of inhibin B and AMH were measured in serum by enzyme immunometric assays (Oxford Bio-Innovation reagents, Serotec, Oxford, UK for inhibin B and Immunotech reagents, Beckman Coulter Company, Marseille, France for AMH). The lower limit of detection was 10 pg/mL for inhibin B and 1 pmol/L for AMH. Their concentrations were compared to the normal values [9,12]

Data are expressed as means ± se. The differences between groups were analyzed by a Kruskall Wallis test, followed by Mann-Whitney tests. Correlations were made with the Spearman rank test. We also analysed the factors associated with abnormal gonadal function using standard statistical tools and Weka Data Mining software [13].

## Results

Gonadal function and the plasma inhibin B concentrations did not differ with the type of HCT (allograft or autograft), or of TBI (single 10 or fractionated 12 Gy). We therefore analysed all the data together.

### 1. Boys

The plasma FSH and LH concentrations indicated that 10 (26%) boys had normal testicular function and 28 (74%) had abnormal function (Table 2). Of the latter, 16 (42%) had isolated tubular failure and 12 (32%) also had Leydig cell failure (11 partial and one complete). The mean plasma inhibin B and AMH concentrations were significantly higher in the boys with normal testicular function than in those with tubular failure, and among these latter higher in those with isolated tubular failure than in those with tubular and Leydig cell failures. Among the 3 boys given HCT after the onset of puberty, the two given TBI and cyclophosphamide had normal testicular function and the one given TBI, melphalan and vincristin had tubular failure.

**Table 2:** Features of testis function after HCT

FSH	Normal	Abnormal
	n (%)	n (%)
Patients	10 (26)	28 (74)

Age at HCT, yr	8.6 ± 1.3	8.1 ± 0.6
Age at evaluation, yr	15.8 ± 0.5	16.7 ± 0.4
Interval between HCT and evaluation, yr	7.3 ± 1.5	8.6 ± 0.6

Conditioning		
TBI	8 (23)	26 (77)
TLI	1 (33)	2 (67)
Chemotheray alone	1	

Cyclophosphamide	+9 (45)	11 (55)
	-1 (5)	17 (95)
Busulfan	+1 (100)	
	-9 (24)	28 (76)
Melphalan	+0	16 (100)
	-10 (45)	12 (55)

Inhibin B, pg/mL	202 ± 58	35 ± 5*
	n = 10	
		Tubular failure, n = 16
		47 ± 7**
		Tubular and Leydig cell failures, n = 12
		20 ± 3
AMH, pmol/L	195 ± 86	41 ± 6***
	n = 8	
		Tubular failure, n = 14
		51 ± 7****
		Tubular and Leydig cell failures, n = 9
		24 ± 6

mean±se

P*<0.0001 compared to normal

P**=0.01 compared to tubular and Leydig cell failures

P***<0.005 compared to normal testis function

P****<0.02 compared to tubular and Leydig cell failure

The 10 boys with normal FSH and LH concentrations included 8 who were given TBI for acute lymphoblastic leukemia (n = 2), acute myeloid leukemia (n = 5), and chronic myelogenous leukemia (n = 1), one who was given TLI for severe aplastic anemia, and one who was given chemotherapy alone (aracytine and busulfan) at one year for acute lymphoblastic leukemia. All, except the 3 treated for acute lymphoblastic leukemia, were given cyclophosphamide alone.

**Figure 1:**
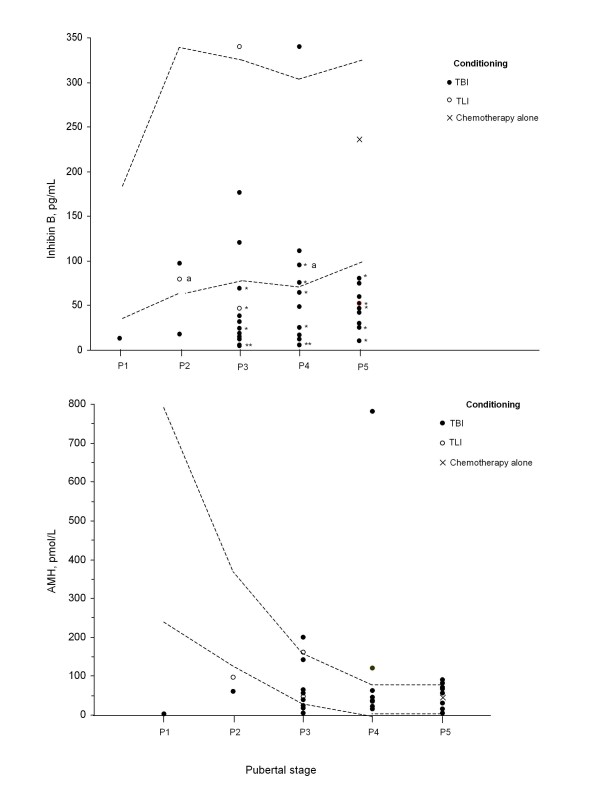
**Distributions of plasma inhibin B and antimüllerian hormone (AMH) in boys given hematopoietic cell transplantation according to the conditioning protocol**. Broken lines to the 5^th ^and 95^th ^percentiles^9,12^; * indicates those given melphalan; ^a ^indicates the 2 boys with increased FSH (10 and 11 IU/L) and normal inhibin B.

All 16 boys who were given melphalan had increased plasma FSH, and 14/16 had decreased inhibin B concentrations (Figure 1). The melphalan was combined with TLI in one case and with TBI in the others (single 10 Gy in four and fractionated 12 Gy in eleven cases).

The plasma AMH concentrations were normal in 25 patients and decreased in 6, all of whom had increased FSH and low inhibin B concentrations (Figure 1). The plasma inhibin B concentrations were normal in 10 and decreased in 28 boys. There were dissociations between the plasma FSH and inhibin B concentrations in 3 patients: one boy with normal FSH had low inhibin B (55 pg/mL); conversely two boys with increased FSH (10 and 11 IU/L) had normal inhibin B (79 and 95 pg/mL). These 3 patients included 2 with testicular volumes greater than 10 mL.

The plasma inhibin B concentrations were positively correlated with those of AMH and negatively correlated with those of FSH (Rho = 0.71, P < 0.0001 for both), but not with the age at HCT, interval between HCT and evaluation, testicular volume, which was negatively correlated with the plasma FSH concentrations (Rho = -0.46, P < 0.006).

Data Mining analysis showed that the factors associated with increased plasma FSH concentrations were melphalan treatment, no cyclophosphamide treatment, and a low inhibin B concentration: all 22 boys with an inhibin B concentration below 55 pg/mL had increased plasma FSH concentrations, while all 7 of the boys whose inhibin B concentration was above 100 pg/mL had normal plasma FSH and LH concentrations.

### 2. Girls

Menarche had occurred spontaneously in 11 girls, 6 of whom had normal FSH and LH concentrations and 5 had partial ovarian failure.

The plasma FSH and LH concentrations indicated that 7 (21%) girls had normal ovarian function and 27 (79%) had ovarian failure (11 partial and 16 complete, Table 3). The mean plasma inhibin B concentrations were significantly higher in the girls with normal ovarian function than in those with ovarian failure, and among these latter higher in those with partial ovarian failure than in those with complete failure. The two girls given HCT after the onset of puberty suffered from ovarian failure.

**Table 3:** Features of ovarian function after HCT

Ovarian function		Normal	Abnormal
		n (%)	n (%)
Patients		7 (21)	27 (79)

Age at HCT, yr		5.3 ± 1.2	7.4 ± 0.7
Age at evaluation, yr		14.7 ± 0.6	13.7 ± 0.3
			
Interval between HCT and evaluation, yr		9.4 ± 0.8	6.3 ± 0.7

Conditioning			
			
TBI		3 (13)	21 (87)
TLI		1 (33)	2 (67)
Chemotheray alone		3 (43)	4 (57)
			
Cyclophosphamide	+	4 (18)	18 (82)
	-	3 (25)	9 (75)
Busulfan	+	1 (12)	7 (88)
	-	6 (23)	20 (77)
Melphalan	+	3 (30)	7 (70)
	-	4 (17)	20 (83)

Inhibin B, pg/mL		87 ± 20	18.6 ± 4.3*
		n = 7	
			Partial ovarian failure, n = 11
			26.9 ± 10ª
			Complete ovarian failure, n = 16
			12.9 ± 1.1
			
AMH, pmol/L		0.7 ± 0.4	0.1 ± 0.1ª
		n = 7	n = 18

mean ± se

a: NS

P* = 0.0003 compared to normal ovarian function

The 7 girls with normal FSH and LH concentrations included 3 who were given TBI for acute lymphoblastic leukemia (n = 2) or acute myeloid leukemia (n = 1), one who was given TLI for severe aplastic anemia and 3 who were given chemotherapy alone for congenital immunodeficiency (= 2) or nephroblastoma.

Among the 8 girls given busulfan, combined with TBI in two, 7 had increased plasma FSH and LH concentrations and 7/8 had low inhibin B with dissociation in one case.

**Figure 2:**
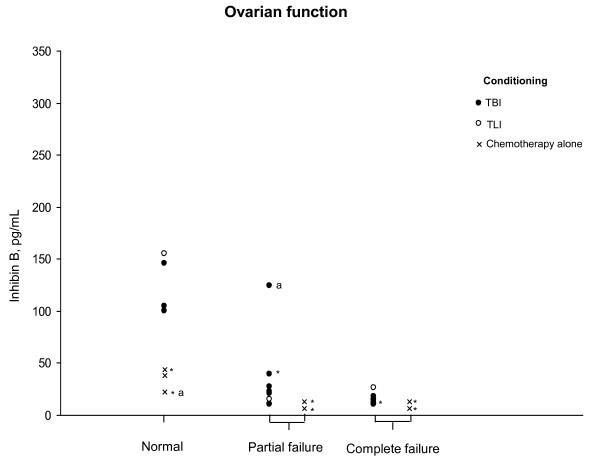
**Distributions of plasma inhibin B in girls given hematopoietic cell transplantation according to the ovarian function and conditioning protocol**. * indicates those given busulfan; ^a ^indicates the 2 girls with dissociations between gonadotropin and inhibin B concentrations.

The plasma AMH concentrations were very low, between 0 and 2 pmol/L, including in the 7 with normal ovarian function. The plasma inhibin B concentrations were normal in 6 and decreased in 28 girls (Figure 2). There were dissociations between the plasma FSH/LH and inhibin B concentrations in 2 patients: one girl given cyclophosphamide and busulfan without irradiation had normal FSH and LH and low inhibin B (22 pg/mL) at 12.2 years; conversely, one girl with increased FSH/LH had normal inhibin B (124 pg/mL) with few spontaneous menstruations.

The plasma inhibin B concentrations were negatively correlated with those of FSH (Rho = -0.6, P = 0.0006), but not with the age at HCT or the interval between HCT and evaluation.

Data Mining analysis showed that the factors associated with increased plasma FSH/LH concentrations were busulfan and a low inhibin B concentration: the inhibin B concentration was below 40 pg/mL in 26 of the 27 girls with increased LH/FSH concentrations, while it was above 40 pg/mL in 6 of the 7 girls (above 100 pg/mL in 5 girls) with normal plasma FSH and LH concentrations.

## Discussion

We have evaluated the specific effect of conditioning for HCT on the gonads. Any patient having other factors that might have interfered with their gonadal function was excluded, except the 27 given HCT in the second or third remission who were previously given chemotherapy without irradiation.

### 1. Boys

Their plasma FSH and LH concentrations indicated that 26% of our boys had normal testicular function. These figures are higher than those reported by Sanders et al [2] (13%) and Van Casteren et al [14] who reported that the 10 boys given TBI 7.5 or 12 Gy before HCT had almost undetectable (< 26 pg/mL, n = 8) or 26-62 pg/mL (n = 2) inhibin B concentrations; the severity of gonadal dysfunction in this study may be partly due to the additional testicular radiotherapy given to 5/10 boys. The differences might also be explained by differences in the age at HCT and/or the interval between HCT and evaluation. However, these two factors did not influence testicular function in our study. The wide ranges of the age at HCT and the interval between HCT and evaluation, and the limited study population might partly explain the absence of any significant impact of these factors in our study.

Melphalan seemed to be more gonadotoxic, giving rise to increased FSH (in all 16) and decreased inhibin B (in 14 of them) concentrations. This effect of melphalan was also seen by Crofton et al [15], who showed that inhibin B was normal before, during and after treatment in 16 boys given chemotherapy for solid tumors or acute lymphoblastic leukemia, except in one who had undetectable inhibin B after cyclophosphamide plus cisplatin plus melphalan. However conclusions cannot easily be drawn as the effect of the drug cannot be separated from that of irradiation.

Our study confirms the excellent correlation and concordance between the plasma concentrations of FSH and inhibin B that was reported by Cicognani et al [16]. They showed that all boys who have increased FSH (> 6.1 IU/L) or LH (> 5.8 IU/L) concentrations also had inhibin B concentrations below 112 pg/mL (-2 standard deviations). Unlike Lähteenmäki et al [17], we found no correlation between testicular volume and inhibin B (P = 0.06). They reported that patients with small testes (< 10 mL) after treatment for childhood malignancy had inhibin B concentrations below 42 pg/mL, and 6 out of 7 had FSH concentrations above 9 IU/L; patients with a testicular volume above 13 mL had inhibin B concentrations above 100 pg/mL. This difference might be explained by variations in the age at which the boys in our study were given HCT and evaluated.

We find that the plasma AMH concentration does not seem to be a good marker, as it normally decreases when testosterone increases at puberty.

We did not carry out a sperm analysis because our boys were too young. However, the reported relationship between inhibin B and the sperm count in normal men and in boys surviving a childhood malignancy suggests that 10/38 (based on FSH or inhibin B) or 7/38 (based on both) of the boys we studied will be normospermic. Thomson et al [18] showed that inhibin B was barely detectable in azoospermic patients whose germ cells had been destroyed, despite preservation of their Sertoli cells, as confirmed by testicular biopsy. Van Beek et al [19] showed that inhibin B is a better marker of spermatogenesis than is the FSH concentration in men given chemotherapy for Hodgkin's lymphoma as children. Multivariate analysis with inhibin B and FSH concentrations as determinants of sperm concentration showed that inhibin B was the only significant determinant; all the men with a plasma inhibin B concentration above 75 pg/mL were normospermic. Lähteenmäki et al [20] showed that the inhibin B and FSH concentrations and the testicular volume explain 44% of the variance in the sperm count. Van Casteren et al [14] found that their 5 patients who were normospermic had inhibin B concentrations of 125-234 pg/mL, while the corresponding values in the 9 azoospermic patients was 0-79 pg/mL, with a correlation between sperm count and inhibin B concentrations.

### 2. Girls

Their plasma FSH and LH concentrations indicate that 21% of our girls had normal ovarian function. These figures are similar to those reported by others [2,21,22]. However, the girls in our study with spontaneous puberty were no younger at HCT than were those with ovarian failure. Similarly, the FSH, inhibin B and AMH concentrations were not influenced by age or pubertal status at treatment. Fong et al [23] studied women treated for cancer during childhood and found that those with undetectable AMH were significantly older at diagnosis, only 2/17 had regular menstrual cycles, and more of them had been given body or abdomen radiotherapy than those with greater AMH concentrations. The preeminence of the role of the irradiation and of busulfan might partly explain why the age at HCT has no significant impact on ovarian function in our study.

Busulfan seemed to be more gonadotoxic. Of the 8 girls given busulfan, combined with TBI in two, 7 had increased plasma FSH/LH concentrations and 7/8 had low inhibin B. This effect of busulfan was also seen by Teinturier et al [24], who showed that the 10 girls given busulfan (600 mg/m^2^) without TBI before autologous HCT all developed severe, persistent ovarian failure. They identified 26 of 29 girls in published reports given busulfan during prepuberty or puberty as having signs of ovarian failure.

Larsen et al [25] used multiple linear regression analysis of 100 girls who had survived childhood malignancy to predict the total number of antral follicles per ovary. Ovarian irradiation, alkylating chemotherapy, older age at diagnosis and a long period off treatment all reduced the number of follicles. Among the 10 girls conditioned for HCT by TBI, the 3 with spontaneous cycles had smaller ovaries, fewer total follicles and higher FSH than the others.

We have therefore confirmed the excellent correlation and concordance between the plasma concentrations of FSH and inhibin B, and agreement with the clinical evolution, while those of AMH were very low. The 7 girls with normal puberty and FSH and LH all had AMH concentrations similar to those with ovarian failure. Three [23,26,27] studies have evaluated the capacity of plasma AMH concentrations to assess the ovarian damage in adults surviving childhood malignancy. Bath et al [26] compared 10 girls treated for cancer during childhood, all with regular menstrual cycles, with 11 controls. The treated girls had smaller ovaries than the controls and significantly higher FSH and lower AMH concentrations, while the other hormone concentrations were unchanged. Thus, the AMH concentration indicates a depletion of ovarian reserve, despite regular menstrual cycles. Van Beek et al [27] showed that girls treated with MOPP (mechlorethamine, oncovin, prednisone, procarbazine) for Hodgkin's lymphoma during childhood developed into women with elevated FSH concentrations and decreased AMH concentrations. Three of the women with normal FSH had decreased AMH.

## Conclusions

In boys, the concordance between plasma FSH and inhibin B concentrations suggests that measuring inhibin B may help in counselling at pubertal age. Plasma AMH concentrations do not seem to be a good marker as they normally decrease when testosterone increases at puberty. All or most of the boys given melphalan, when combined with TBI or TLI, will be azoospermic.

In girls, there is also a good concordance between plasma FSH and inhibin B concentrations. Both were normal in 6/34 girls who had normal puberty and regular menstruations, but their very low AMH concentrations suggest that there is a major loss of primordial follicles.

One limitation of this study is the range of drugs used. The fact that the drug effect could not be separated from that of irradiation significantly limits our ability to draw any conclusion about the effects of melphalan or busulfan.

## Abbreviations

AMH: antimüllerian hormone; FSH: follicle-stimulating hormone, growth hormone - GH; Gy: Grays; HCT: hematopoietic cell transplantation; LH: luteinizing hormone; TLI: total lymphoid irradiation; TBI: total body irradiation

## Competing interests

The authors declare that they have no competing interests.

## Authors' contributions

SL and AC-CS participated in the design of the study, data acquisition and analysis. ST and SBT carried out the immunoassays. PL and CT performed the statistical analyses. CT prepared the tables and the figures. HE, JM, AB and AF followed the patients and participated in the design of the study. RB directed the work and prepared the manuscript. All authors read and approved the final manuscript.

## Pre-publication history

The pre-publication history for this paper can be accessed here:

http://www.biomedcentral.com/1471-2431/11/20/prepub
